# Epigenetic regulation of *CD44 *in Hodgkin and non-Hodgkin lymphoma

**DOI:** 10.1186/1471-2407-10-517

**Published:** 2010-09-29

**Authors:** Sonja Eberth, Björn Schneider, Andreas Rosenwald, Elena M Hartmann, Julia Romani, Margarete Zaborski, Reiner Siebert, Hans G Drexler, Hilmar Quentmeier

**Affiliations:** 1 DSMZ - German Collection of Microorganisms and Cell Cultures, Braunschweig, Germany; 2 Institute of Pathology, University of Würzburg, Würzburg, Germany; 3 Institute of Human Genetics, Christian-Albrechts University, Kiel, Germany

## Abstract

**Background:**

Epigenetic inactivation of tumor suppressor genes (TSG) by promoter CpG island hypermethylation is a hallmark of cancer. To assay its extent in human lymphoma, methylation of 24 TSG was analyzed in lymphoma-derived cell lines as well as in patient samples.

**Methods:**

We screened for TSG methylation using methylation-specific multiplex ligation-dependent probe amplification (MS-MLPA) in 40 lymphoma-derived cell lines representing anaplastic large cell lymphoma, Burkitt lymphoma (BL), diffuse large B-cell lymphoma (DLBCL), follicular lymphoma (FL), Hodgkin lymphoma and mantle cell lymphoma (MCL) as well as in 50 primary lymphoma samples. The methylation status of differentially methylated *CD44 *was verified by methylation-specific PCR and bisulfite sequencing. Gene expression of *CD44 *and its reactivation by DNA demethylation was determined by quantitative real-time PCR and on the protein level by flow cytometry. Induction of apoptosis by anti-CD44 antibody was analyzed by annexin-V/PI staining and flow cytometry.

**Results:**

On average 8 ± 2.8 of 24 TSG were methylated per lymphoma cell line and 2.4 ± 2 of 24 TSG in primary lymphomas, whereas 0/24 TSG were methylated in tonsils and blood mononuclear cells from healthy donors. Notably, we identified that *CD44 *was hypermethylated and transcriptionally silenced in all BL and most FL and DLBCL cell lines, but was usually unmethylated and expressed in MCL cell lines. Concordant results were obtained from primary lymphoma material: *CD44 *was not methylated in MCL patients (0/11) whereas *CD44 *was frequently hypermethylated in BL patients (18/29). In cell lines with *CD44 *hypermethylation, expression was re-inducible at mRNA and protein levels by treatment with the DNA demethylating agent 5-Aza-2'-deoxycytidine, confirming epigenetic regulation of *CD44*. CD44 ligation assays with a monoclonal anti-CD44 antibody showed that CD44 can mediate apoptosis in CD44^+ ^lymphoma cells. *CD44 *hypermethylated, CD44^- ^lymphoma cell lines were consistently resistant towards anti-CD44 induced apoptosis.

**Conclusion:**

Our data show that *CD44 *is epigenetically regulated in lymphoma and undergoes *de novo *methylation in distinct lymphoma subtypes like BL. Thus *CD44 *may be a promising new epigenetic marker for diagnosis and a potential therapeutic target for the treatment of specific lymphoma subtypes.

## Background

Cancer cells display multiple defects in cellular pathways that govern normal cellular proliferation and homeostasis. During their development cancer cells acquire a set of functional capabilities for malignant growth, usually including self-sufficiency in growth signals, insensitivity to growth-inhibitory signals, evasion from apoptosis, limitless replicative potential, sustained angiogenesis, and tissue invasion and metastasis [1]. These essential alterations in cell physiology are, amongst others, achieved by the constitutive activation of oncogenes and the loss of tumor suppressor gene (TSG) function. Both, genetic and epigenetic mechanisms contribute to the inactivation of TSG. Genetic alterations often include deletions and loss-of-function mutations. Furthermore, TSG may become epigenetically silenced by hypermethylation of CpG islands located in their promoter regions, which are usually unmethylated in normal tissue [2,3]. Cytosine methylation of CpG dinucleotides is catalyzed by DNA methyltransferases [4]. DNA methylation interferes with binding of transcription factors and, additionally, methylated CpG are bound by methyl-CpG binding proteins that induce the formation of inactive chromatin by interacting with histone deacetylases, resulting in transcriptional repression [2,5]. Epigenetic silencing of TSG is potentially reversible. Thus, hypermethylated TSG promoters represent therapeutic targets for DNA demethylating agents like 5-Aza-2'-deoxycytidine (Aza, Decitabine), as already demonstrated in clinical trials [6].

TSG hypermethylation in cancer cells has strong specificity with respect to the tissue of origin and tumor-type-specific methylation patterns have been reported for primary human tumors and cancer cell lines [7-11]. Several studies showed differential DNA methylation patterns and discovered subtype-specific epigenetic markers in diverse lymphoid malignancies [12-15]. Furthermore, hypermethylation of certain TSG can be correlated with prognosis [16-18]. Yet, functional and genetic studies are necessary for the determination of lymphomagenesis-relevant hypermethylation events.

We aimed to analyze TSG methylation patterns in a broad spectrum of Hodgkin lymphoma-(HL) and non-Hodgkin lymphoma-(NHL) derived cell lines and primary samples to identify novel characteristic methylation patterns of TSG. Besides the discovery of generally hypermethylated TSG, we identified TSG with subtype-specific methylation patterns. One of these novel candidates was *CD44*, formerly described as lymphocyte homing receptor [19]. *CD44 *hypermethylation resulting in gene silencing has already been reported for other cancer types like prostate cancer and neuroblastoma [20-22]. Recently, *CD44 *has been shown to be a novel translocation partner of *IGH *in mature B-cell malignancies resulting in overexpression of *CD44 *lacking exon 1 [23].

We show here, that *CD44 *was frequently hypermethylated and transcriptionally silenced in anaplastic large cell lymphoma (ALCL), Burkitt lymphoma (BL), diffuse large B-cell lymphoma (DLBCL) and follicular lymphoma (FL) cell lines, whereas it was unmethylated and expressed in most HL and mantle cell lymphoma (MCL) cell lines. Importantly, this methylation and expression pattern resembled that of primary lymphomas. Experiments with a DNA demethylating agent verified epigenetic regulation of *CD44*. Furthermore, *CD44 *hypermethylated cell lines were resistant towards anti-CD44 induced apoptosis. This supports a tumor suppressor role for CD44 in lymphoma and highlights *CD44 *as an interesting new potential epigenetic marker and a valuable target for epigenetic therapy in distinct lymphoma subtypes.

## Methods

### Human cell lines

Cell lines were either resourced from the DSMZ cell repository (Braunschweig, Germany) or were generously provided by original investigators. Detailed references and cultivation protocols have been described previously [24].

### Samples and patients

DNA was extracted from tumor containing tissues diagnosed or suspicious for Burkitt lymphoma/leukemia, in which chromosome analysis revealed the presence for a Burkitt translocation t(8;14) or variants, and from frozen sections of DLBCL and MCL from untreated patients using the DNeasy Mini Kit (Qiagen, Hilden, Germany). The study on BL material was performed according to the protocols of the network "Molecular Mechanisms in Malignant Lymphoma" for which approval by the Ethics Committee of the Medical Faculty of the Christian-Albrechts-University Kiel was obtained. The study on DLBCL and MCL material was approved by the local Ethics Committee of the Medical Faculty of the University of Würzburg.

### Methylation-specific multiplex ligation-dependent probe amplification (MS-MLPA)

The MS-MLPA assay (ME001B; MRC-Holland, Amsterdam, Netherlands) simultaneously detects copy number changes and CpG methylation of the promoter regions of 24 different TSG. This semi-quantitative technique is based on digestion of DNA with the methylation-sensitive restriction enzyme *Hha*I (Fermentas, St. Leon-Rot, Germany) and a subsequent multiplex PCR followed by fragment analysis via capillary electrophoresis [25]. MS-MLPA data were analyzed using a Microsoft Excel spreadsheet from the National Genetics Reference Laboratory designed specifically for the ME001B assay http://www.ngrl.org.uk/Manchester/projects/informatics/mlpa. Levels of methylation were calculated by comparing relative peak areas of the *Hha*I digested ligation products with the corresponding ligation products from undigested samples. Peak areas were normalized relative to neighboring control ligation products prior to comparison. Samples with a methylation level > 10% were regarded as methylated.

### Aza treatment for demethylation of DNA

5-Aza-2'-deoxycytidine (Sigma-Aldrich, Taufkirchen, Germany) dissolved in DMSO was used to verify the effect of promoter methylation on TSG expression. Cells were seeded at a density of 5 × 10^5^/ml, Aza was added at a final concentration of 5 μM. Vehicle control cells were treated with 0.05% DMSO. After 2 days, half of the medium was replenished with medium with/without Aza (5 μM). After 3 or 4 days, cells were harvested to prepare RNA or used for CD44 detection by flow cytometry.

### RNA isolation and quantitative real-time PCR

RNA was isolated using the RNeasy Mini Kit (Qiagen) including DNase digestion. Reverse transcription was performed with SuperScript II reverse transcriptase Kit (Invitrogen, Karlsruhe, Germany). qRT-PCR for gene expression analysis was performed on an Applied Biosystems 7500 SDS real-time PCR system (Darmstadt, Germany). Expression of *RPS9 *as endogenous control and of *CD44 *and *CD44v6 *was assessed using SYTO-82 (Molecular Probes, Leiden, Netherlands) as fluorescent dye and ImmoMix (Bioline, Luckenwalde, Germany) as PCR master mix. Primers were the following: *CD44 *fwd 5'-CAA TAG CAC CTT GCC CAC AAT-3', *CD44 *rev 5'-AAT CAC CAC GTG CCC TTC TAT GG-3', *CD44v6 *fwd 5'-GAT CAC CGA CAG CAC AGA CA-3', *CD44v6 *rev 5'-CCA TCT GTT GCC AAA CCA CTG-3', *RPS9 *fwd 5'-GGG AAG CGG AGC CAA CAT G-3', *RPS9 *rev 5'-GTT TGT TCC GGA GCC CAT ACT-3'. Relative gene expression levels were calculated using the ΔΔCt-method.

### Bisulfite conversion and methylation-specific PCR (MSP)

Bisulfite conversion of DNA was performed as described by the supplier (EpiTect Bisulfite Kit, Qiagen). For methylation analysis of the *CD44 *(ENSEMBL ID ENSG00000026508) exon 1 region, including the regulatory promoter region described by Shtivelman and Bishop [26], we performed nested PCR with first-round primers CD44 BSP fwd 5'-GGT TGA ATT TAA TGG TGT AAG G-3' and CD44 BSP rev 5'-ACA ACT CAC TTA ACT CCA ATC C-3' amplifying bisulfite-converted DNA independently of the *CD44 *methylation status (bisulfite-specific PCR, BSP), while second-round primers for M-PCR CD44 M_fwd 5'-TCG TTG AGT TTG GCG TAG ATC-3', CD44 M_rev 5'-ACT ACC GCC GAA TCC GCG-3' and for U-PCR CD44 U_fwd 5'-GTG TTG TTG AGT TTG GTG TAG ATT-3', CD44 U_rev 5'-CAA AAA AAC TAC CAC CAA ATC CAC A-3' specifically recognized the methylated or unmethylated versions of the region. PCR products of the initial BSP were diluted 1:100 for subsequent MSP. Epitect PCR Control DNA (Qiagen) was used as control for methylated and unmethylated templates.

### Bisulfite sequencing

For bisulfite sequencing of the *CD44 *exon 1 region, *CD44*-BSP products obtained with primers CD44 BSP fwd and CD44 BSP rev (sequences see previous section), were purified and subcloned into the pGEM-Teasy plasmid vector (Promega, Mannheim, Germany). Plasmid DNA from at least eight insert-positive clones for each sample was isolated using QIAprep Spin Miniprep Kit (Qiagen) and subjected to automated sequencing. Sequences were evaluated using BiQ Analyzer http://biq-analyzer.bioinf.mpi-sb.mpg.de[27] and had to conform to at least 90% bisulfite conversion rate. Identical clones were excluded from the analysis as well.

### Analysis of CD44 protein expression by flow cytometry

For detection of CD44 on the cell surface, cells were washed and incubated with mouse anti-human CD44 mAB (clone G44-26, BD Biosciences, Heidelberg, Germany) or with the isotope-matched control mouse immunoglobulin (BD Biosciences) for 30 min at 4°C. Subsequently, cells were treated with FITC conjugated anti-mouse secondary antibody (Biozol, Eching, Germany) and Propidium Iodide (PI) (Sigma-Aldrich). Labeled cells were analyzed on a FACSCalibur (BD Biosciences) using CellQuest Pro software.

### CD44 splice variant analysis

For the analysis of *CD44 *splice variants, reverse transcriptase PCR was performed using *CD44 *primers flanking all variant exons: CD44 exon 5 s fwd 5'-CAG ACA GAA TCC CTG CTA CCA-3', CD44 Exon 7 s rev 5'-GTC CTT ATA GGA CCA GAG GTT G-3'. The PCR reaction mixture was cycled for 30 s at 94°C, 30 s at 62°C, and 1 min at 72°C for 40 cycles. The PCR product of *CD44 s *corresponds to 142 bp. For sequencing of unknown splice variants, PCR products were sliced from agarose gel, purified with QIAquick Gel Extraction Kit (Qiagen) and subcloned into the pGEM-Teasy plasmid vector (Promega). Plasmid DNA was isolated using QIAprep Spin Miniprep Kit (Qiagen) and subjected to automated sequencing.

### CD44 ligation and annexin-V staining

Cells were seeded at 10^6^/ml in 96-well tissue culture plates and cultured in the presence of anti-CD44 mAb (clone G44-26, BD Biosciences) or the isotope-matched control mouse immunoglobulin (BD Biosciences) at a concentration of 5 μg/ml or 20 μg/ml for 24 h. Cells were then processed for apoptosis studies via annexin-V staining using the TACS Annexin-V-FITC Kit (R&D Systems, Wiesbaden, Germany) according to the manufacturer's instructions. Cells were analyzed by flow cytometry on a FACSCalibur (BD Biosciences) using CellQuest Pro software.

## Results and Discussion

### Screening for tumor suppressor gene methylation with MS-MLPA

**Figure 1:**
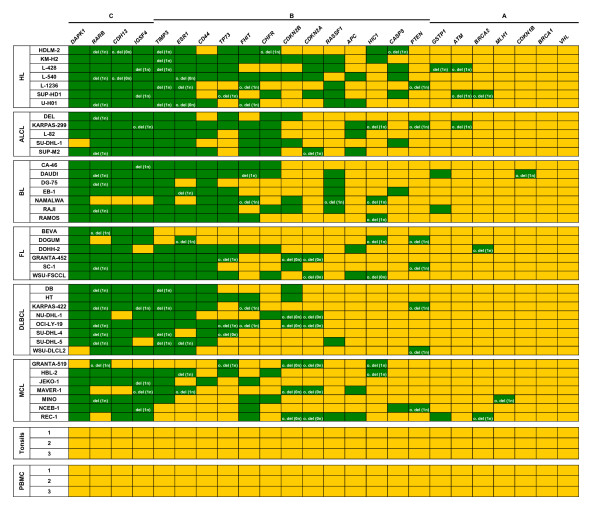
**Methylation status and deletions of TSG in lymphoma cell lines**. MS-MLPA results of 40 lymphoma cell lines show the methylation status and copy number of 24 TSG. As control, DNA samples from tonsils and PBMC of three healthy donors were analyzed. In each lymphoma subgroup cell lines are arranged alphabetically. TSG are sorted from left to right according to frequency of methylation and/or deletion. According to the incidences of methylated and/or deleted TSG in cell lines, TSG can be divided in 3 groups: group A (methylated and/or deleted in ≤ 10% of cell lines), group B (methylated and/or deleted in 10 to 89% of cell lines) and group C (methylated and/or deleted in ≥ 90%). yellow = TSG unmethylated and not deleted; green = TSG methylated and/or deleted; del = deleted; o. del = only deleted, not methylated.

Besides gene deletions, TSG are a frequent subject of promoter hypermethylation in tumor cells. Both types of alteration can result in functional loss of a TSG. To quantify epigenetic and genetic changes of 24 TSG, we used the semi-quantitative MS-MLPA assay. We screened a panel of 40 lymphoma cell lines (Figure 1) representing HL and five distinct subtypes of NHL (ALCL, BL, DLBCL, FL and MCL) and 50 primary lymphoma samples from BL, DLBCL and MCL patients (Additional File 1) to identify TSG methylation patterns and TSG deletions. As expected, control DNA of tonsils and peripheral blood mononuclear cells (PBMC) from three healthy donors showed no methylation or deletion of any TSG (Figure 1).

On average 8 ± 2.8 of 24 TSG were methylated per lymphoma cell line. TSG deletions were less frequent with an average of 2.4 ± 1.5 per cell line. The loss of one allele and methylation of the second one could be observed frequently in lymphoma cell lines for *RARB *(*retinoic acid receptor beta*) and to a lesser extent for *TIMP3 *(*tissue inhibitor of metalloproteinase 3*), *IGSF4 *(*immunoglobulin superfamily member 4*, alias *CADM1*) and *ESR1 *(*estrogen receptor 1*) (Figure 1). The examination of primary material from 29 BL, 10 DLBCL and 11 MCL patients with MS-MLPA showed that the average number of methylated TSG in primary material was lower (2.4 ± 2 of 24) than in cell lines (Additional File 1). The higher level of CpG island hypermethylation identified in cell lines in comparison to the primary tumors has also been reported by others [28]. However, although cancer cell lines have an increased rate of hypermethylation of certain CpG islands, they retain the specific CpG island hypermethylation profile of each tumor cell [9,29]. Consistently, the most frequently methylated TSG were the same in cell lines and primary samples: *DAPK1 *(*death-associated protein kinase 1*), *RARB*, *CDH13 (cadherin 13)*, *IGSF4*, *TIMP3*, *ESR1 *and *CD44*.

The presence of generally and differentially methylated TSG among the lymphoma subtypes analyzed is in line with previous reports describing the existence of genes commonly hypermethylated in various tumors and genes being epigenetically silenced in a subtype-specific manner [7,15]. Some of the observed TSG methylation patterns in lymphoma cell lines (e.g. for *DAPK1*, *RASSF1*) support previous methylation analyses performed on primary lymphoma patient material [30-33]. In addition, the MS-MLPA screening in lymphoma cell lines and primary samples led to the identification of novel TSG methylation profiles for *RARB*, *TIMP3*, *CDH13*, *IGSF4 *and *ESR1 *which were frequently methylated in lymphoma (Figure 1, Additional File 1).

From our point of view *CD44 *showed the most interesting and hitherto unknown methylation pattern: it was methylated in all BL cell lines (7/7) but not methylated in most of the MCL cell lines (1/7) (Figure 1). Furthermore, *CD44 *methylation was detected in 9/29 BL patient samples but not in any of the MCL patients (0/11) (Additional File 1). To our knowledge there are no reports on the potentially epigenetic regulation of *CD44 *in lymphoma cells.

### Verification of differential *CD44 *methylation in lymphoma

**Figure 2:**
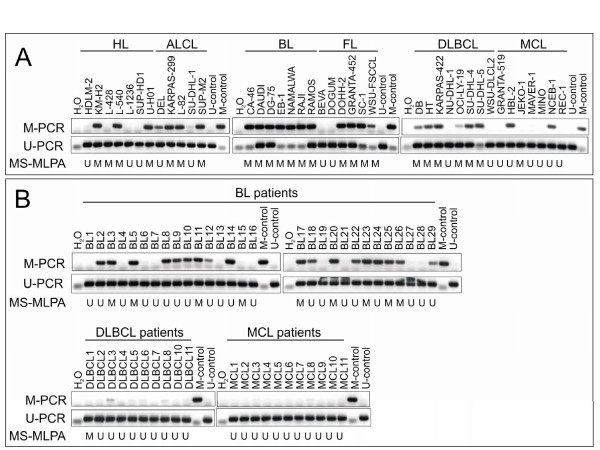
**Methylation status of *CD44 *in 40 lymphoma cell lines and 50 patient samples according to MSP**. *CD44 *methylation in lymphoma cell lines **(A) **and material from BL, DLBCL and MCL patients **(B) **was analyzed by MSP after bisulfite conversion of the DNA. *CD44 *MSP agarose gels of M-and U-PCR are shown. The *CD44 *methylation status as determined by MS-MLPA is indicated underneath: M = sample methylated, U = sample unmethylated.

**Figure 3:**
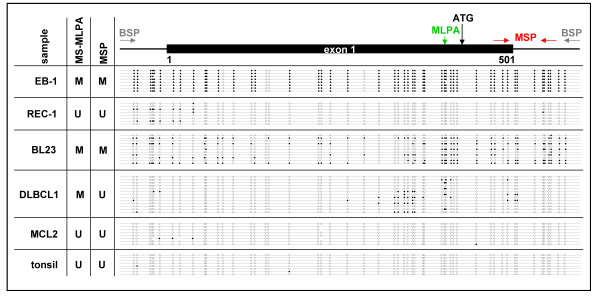
**Bisulfite sequencing of *CD44 *exon 1 region**. The CpG island of *CD44 *is located between-638 and +496 relative to the ATG codon, thereby spanning the whole exon 1. The *CD44 *exon 1 region (682 bp, 53 CpG sites) was sequenced after bisulfite conversion of DNA from cell line EB-1 (BL) and cell line REC-1 (MCL) as well as from patient samples BL23 (BL), DLBCL1 (DLBCL) and MCL2 (MCL) and tonsil DNA from a healthy donor. Each line depicts a sequenced clone representing the methylation status of an individual allele. CpGs are represented as open dots (if unmethylated) or filled dots (if methylated). In BL cell line EB-1 and in the BL patient clones with dense CpG methylation were detected, the DLBCL patient showed partial CpG methylation next to the ATG codon. In contrast no methylation of these sites was detectable in the MCL cell line REC-1, the MCL patient and normal tonsil DNA. Bisulfite sequencing confirmed results of *CD44 *methylation status detected with MS-MLPA and MSP. Note that CpG sites analyzed by the MS-MLPA probe (green) and MSP primers (red) are not identical.

The MS-MLPA assay result is based on the methylation status of only one single CpG site in the context of an *Hha*I restriction site in the promoter region of a gene, rendering it suitable as a screening-method [25]. To assess the significance of the *CD44 *methylation status obtained by MS-MLPA, we analyzed *CD44 *methylation with additional independent techniques: methylation-specific PCR (MSP) and bisulfite sequencing (Figures 2 and 3). After bisulfite conversion of DNA we determined the methylation status of *CD44 *with MSP in the lymphoma cell lines (Figure 2A) and primary samples (Figure 2B). According to MSP *CD44 *was methylated in all (7/7) BL cell lines, frequently methylated in ALCL (4/5), FL (4/6) and DLBCL (6/8) cell lines, and to a lesser extent methylated in HL (3/7) and MCL (2/7) cell lines, which matched the MS-MLPA results (Figure 2A). In accordance with the *CD44 *methylation pattern observed in cell lines, *CD44 *was frequently methylated in BL patients (18/29) but not methylated in MCL patients (0/11) (Figure 2B). In contrast only one DLBCL patient (1/10) showed *CD44 *methylation (Figure 2B), thus *CD44 *methylation occurred less frequently in DLBCL patients than in DLBCL cell lines. Overall, the results of MSP and MS-MLPA showed good concordance in cell lines (87.5%) and primary samples (74%) as well. In particular cases the different results of MS-MLPA and MSP might be the consequence of distinct CpG sites analyzed. Additionally, MSP is more sensitive than MS-MLPA.

Therefore, the methylation status of *CD44 *in lymphoma cell lines and primary samples was finally verified by bisulfite sequencing of the CpG island spanning exon 1 of *CD44*. Sequencing confirmed dense CpG methylation of *CD44 *in the BL cell line EB-1, which was *CD44 *hypermethylated according to MS-MLPA and MSP (Figure 3). In line with MS-MLPA and MSP results, the MCL cell line REC-1 showed no hypermethylation of the exon 1 region of *CD44 *(Figure 3). Also in primary samples bisulfite sequencing verified the MSP results: BL patient BL23 harbored clones with dense CpG methylation in the exon 1 region of *CD44*, whereas MCL patient MCL2 was not methylated at nearly all CpG sites analyzed. DLBCL patient DLBCL1 showed only partial methylation of the CpG sites next to the ATG codon. Furthermore, tonsil DNA of a healthy donor had a completely unmethylated *CD44 *exon 1 region (Figure 3). Thus, *CD44 *might in fact represent a TSG undergoing *de novo *methylation in distinct lymphoma subtypes like BL.

### *CD44*: a novel epigenetically regulated TSG in lymphoma

**Figure 4:**
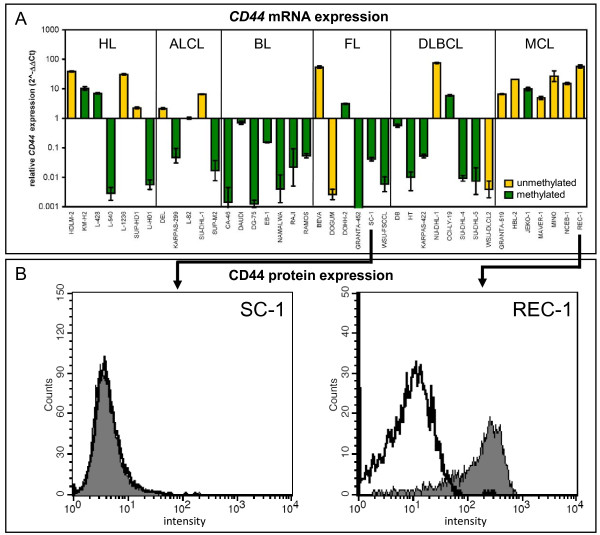
**Correlation between *CD44 *methylation and gene silencing**. **(A) **Transcript levels of *CD44 *were analyzed by qRT-PCR in 40 lymphoma cell lines of the different lymphoma subtypes. *RPS9 *expression was used as endogenous control and cell line L-82 was used for normalization. *CD44 *was primarily expressed in HL and MCL cell lines. Note the strong correlation between *CD44 *methylation (green columns) and transcriptional silencing as well as *CD44 *non-methylation (yellow columns) and *CD44 *expression. The methylation status is shown according to MS-MLPA analysis. **(B) **CD44 protein expression was analyzed in the *CD44 *methylated and mRNA-negative cell line SC-1 (FL) and in the *CD44 *unmethylated, mRNA-positive cell line REC-1 (MCL) by flow cytometry. In accordance with mRNA expression, only cell line REC-1 was positive for CD44 protein. CD44 stained cells (filled histogram) and isotope control labeled cells (open histogram) are shown.

Methylation of TSG has biological relevance if hypermethylation of the promoter region inhibits gene expression. To evaluate the correlation between methylation of the *CD44 *exon 1 region and *CD44 *transcription we performed quantitative real-time PCR (qRT-PCR) with cDNA from lymphoma cell lines. *CD44 *was expressed in all (7/7) MCL, most (5/7) HL and some (3/5) ALCL cell lines, but rarely transcribed in BL, FL and DLBCL cell lines (Figure 4A). In the majority of the lymphoma cell lines (80%), *CD44 *gene expression was inversely correlated with *CD44 *hypermethylation as highlighted by the color of the columns (Figure 4A). This is a remarkable correlation and suggests that *CD44 *is indeed regulated by DNA methylation in lymphoma cells.

Next, we investigated whether *CD44 *hypermethylation was also inversely correlated with CD44 protein expression. Cell surface CD44 protein expression was analyzed by flow cytometry with anti-CD44 (G44-26) monoclonal antibody (mAb) directed against epitope 1, recognizing all forms of CD44 [34]. CD44 protein was expressed on lymphoma cell lines, which were positive for *CD44 *mRNA and predominantly unmethylated in the *CD44 *exon 1 region, especially in MCL and HL cell lines. Cell lines with *CD44 *hypermethylation were negative for *CD44 *mRNA and CD44 protein (Table 1, Figure 4B). Thus, *CD44 *hypermethylation was inversely correlated with gene transcription and protein expression in lymphoma cell lines.

**Table 1:** *CD44 *methylation status, mRNA and protein expression in lymphoma cell lines

subtype	cell line	MS-MLPA	MSP	*CD44 *mRNA	Aza induction of *CD44 *mRNA	CD44 protein
HL	KM-H2	M	M/U	positive	-	positive
	L-428	M	U	positive	n.d.	positive
	L-1236	U	U	positive	n.d.	n.d.
	SUP-HD1	U	U	positive	n.d.	positive

ALCL	DEL	U	M/U	positive	n.d.	positive
	KARPAS-299	M	M/U	negative	+++	negative

BL	CA-46	M	M	negative	+++	negative
	DAUDI	M	M/U	negative	+	n.d.
	EB-1	M	M	negative	++	negative
	RAJI	M	M	negative	+++	negative

FL	DOGUM	U	U	negative	-	n.d.
	DOHH-2	M	M/U	positive	-	n.d.
	SC-1	M	M/U	negative	+++	negative
	WSU-FSCCL	M	M	negative	++	n.d.

DLBCL	DB	M	M/U	negative	+	n.d.
	HT	M	M/U	negative	++	negative
	SU-DHL-4	M	M/U	negative	n.d.	negative
	WSU-DLCL2	U	U	negative	-	negative

MCL	GRANTA-519	U	U	positive	-	positive
	HBL-2	U	M/U	positive	n.d.	positive
	JEKO-1	M	U	positive	-	positive
	REC-1	U	U	positive	-	positive

Gene expression and induction of *CD44 *mRNA transcription were analyzed by qRT-PCR. Aza induction was tested after incubation of cells for 3 days with 5 μM Aza. CD44 protein expression was analyzed by flow cytometry. MS-MLPA and MSP results: M: methylated, U: unmethylated; fold of Aza induction: + > 3 ×, ++ > 30 ×, +++ > 150 ×, - = no change; n.d.: not determined.

**Figure 5:**
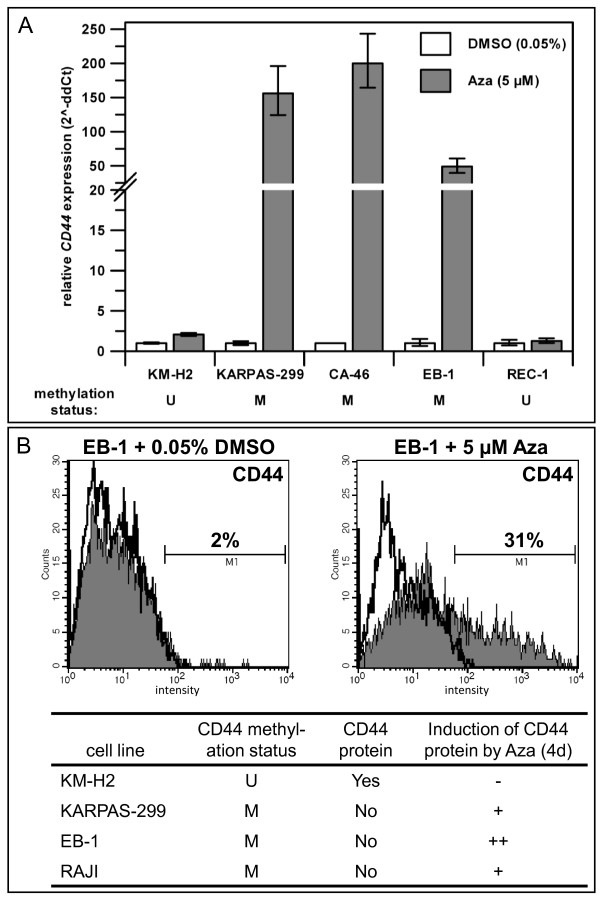
**Effect of the DNA demethylating agent Aza on *CD44 *expression**. **(A) **Expression of *CD44 *was re-inducible by treatment of cells with 5 μM Aza (3 days) in *CD44 *hypermethylated cell lines, but not in *CD44 *unmethylated cell lines as assessed by qRT-PCR. Expression levels of the corresponding DMSO-controls were set to 1. **(B) **Aza (5 μM) treatment for 4 days induced CD44 protein expression in *CD44 *hypermethylated cell lines as analyzed by flow cytometry. CD44 stained cells (filled histogram) and isotope control labeled cells (open histogram) are shown. M: methylated; U: unmethylated; increase of CD44 positive cells: - = no change, + = > 10%, ++ = > 25%.

To test whether *CD44 *expression is epigenetically regulated via promoter methylation in lymphoma, we treated cell lines with Aza, leading to DNA demethylation. The results confirmed that hypermethylation of *CD44 *was responsible for gene silencing since DNA demethylation resulted in reactivation of *CD44 *transcription in *CD44 *hypermethylated cell lines, but not in *CD44 *unmethylated cell lines as determined by qRT-PCR (Figure 5A, Table 1). Furthermore, Aza treatment resulted in induction of CD44 protein expression as shown for cell lines KARPAS-299 (ALCL), EB-1 (BL) and RAJI (BL) by flow cytometry (Figure 5B). The effect of Aza on methylated *CD44 *seemed to be direct since two cell lines (DOGUM and WSU-DLCL2) which were negative for *CD44 *despite being unmethylated, remained *CD44 *negative after Aza treatment (Table 1). However, these results show that DNA methylation is not the only reason for *CD44 *silencing and other suppressive mechanisms appear to play a role in DOGUM and WSU-DLCL2. It has been reported that BCL-6 and p53 are repressors of *CD44 *[35,36]. In breast cancer *CD44 *can be suppressed by miR-373 and miR-520c [37]. Alternatively, essential transcriptional activators might be missing in the CD44^- ^and *CD44 *unmethylated cell lines. In accordance with this view, the *CD44 *promoter is reportedly stimulated by growth factors, particularly by the Ras-Erk signaling pathway [38]. Interesting in this context is also that hypermethylated *CD44 *could be reactivated not only by Aza but also by cAMP in an ATRA-resistant acute promyelocytic leukemia cell line [39]. Thus DNA methylation is one important but not exclusive mechanism regulating the expression of *CD44*.

Significantly, CD44 protein expression data from primary lymphoid material reported by others resemble the CD44 expression pattern, which we observed in lymphoma cell lines (Table 2). The standard CD44 isoform CD44s, skipping all variant exons, is mainly expressed in hematopoietic cells and shows high expression in normal lymphoid tissue [40-42]. Already hematopoietic progenitor cells display positivity for CD44 [43]. In line with this, we identified no *CD44 *methylation in PBMC and tonsils of healthy donors thus enabling transcription of *CD44 *in these cells (Figures 1 and 3). Thus, lymphomas with an unmethylated *CD44 *regulatory region and expressing CD44s reflect their normal lymphocytic counterparts. MCL patient samples are characteristically positive for CD44s [44]. This is in agreement with our data on MCL cell lines, showing *CD44 *expression and an unmethylated *CD44 *promoter (Table 2). Moreover, we detected no *CD44 *hypermethylation in MCL patients (Figure 2B, Table 2). Lymphoma cells of HL and ALCL patients have been described to be often CD44 positive [45,46]. Consistent with these data, HL, and to a lesser extent ALCL cell lines expressed *CD44 *and had an unmethylated *CD44 *exon 1 region (Table 2). CD44 positive and negative cases have been reported for DLBCL patients and it was shown that CD44s expression is inversely correlated with clinical disease stage [47]. However, the majority of DLBCL cell lines did not express *CD44 *due to promoter hypermethylation (Table 2). In NHL patients with BL or nodal FL CD44 is typically absent [45,48]. In accordance with these reports, *CD44 *was not expressed and hypermethylated in all BL and most of the FL cell lines (Table 2). Furthermore, we identified frequent hypermethylation of *CD44 *in BL patients (Figure 2B, Table 2), implying that DNA methylation is the reason for *CD44 *silencing also in primary BL.

**Table 2:** CD44s expression in lymphoma: comparison of published data with this study

	published data	this study		
	
Lymphoma subtype	CD44s protein [reference]	*CD44 *unmethylated promoter in primary lymphomas	*CD44 *unmethylated promoter in cell lines	CD44s expression in cell lines
HL	pos [45]	n.d.	3/7	5/7
ALCL	pos [46]	n.d.	2/5	3/5
BL	neg [48,55]	11/29	0/7	0/7
FL nodal FL	pos/neg [55] neg [45]	n.d.	2/6	2/6
DLBCL	pos/neg [47,55,56]	9/10	2/8	2/8
MCL	pos [44]	11/11	6/7	7/7
normal lymphoid tissue	pos [40,41]	3/3		

Comparison of CD44s protein expression in samples of lymphoma patients as reported in the literature and *CD44 *methylation status and *CD44s *gene expression in lymphoma cell lines and patient samples analyzed in our study. *CD44 *methylation status was determined by MSP (primary samples) or MS-MLPA (cell lines), *CD44 *gene expression levels were analyzed by qRT-PCR. pos = most of the reported samples were CD44 positive, neg = all analyzed samples were negative for CD44. pos/neg = CD44^+ ^and CD44^- ^samples were described, n.d. = not determined.

In conclusion, we propose that *CD44 *expression is regulated by DNA methylation not only in lymphoma cell lines, but also in the primary tumors. And we suppose that the loss of CD44 mRNA and protein expression in distinct lymphoma subtypes is the consequence of *CD44 de novo *methylation.

### CD44 splice variant analysis

Depending on the cell type and the expression of *CD44 *splice variants CD44 proteins can have both oncogenic or tumor suppressor functions [38]. In some hematological malignancies expression of CD44 variants is prognostically significant. Notably, expression of the variant isoform CD44v6 is associated with shorter overall survival in patients with aggressive NHL [42]. Therefore, we analyzed expression of *CD44v6 *in CD44^+ ^lymphoma cell lines with qRT-PCR. However, none of the 15 analyzed cell lines expressed the metastases-associated *CD44v6 *isoform (data not shown). Since we could not exclude the expression of any other variant isoform from these data alone, we investigated *CD44 *transcripts obtained by RT-PCR with primers flanking the variant exons of *CD44 *(Additional File 2). All CD44^+ ^lymphoma cell lines and PBMC had the *CD44s *transcript. Sequencing of the other PCR product obtained revealed that *CD44v10 *was expressed in most of the CD44^+ ^cell lines and PBMC, but to a significantly lesser extent than the standard isoform *CD44s *(Additional File 2). Interestingly, expression of CD44v10 has been reported for Hodgkin and Reed-Sternberg cells in nodular sclerosis HL patients and was associated with an unfavorable prognosis [49]. However, *CD44v10 *mRNA is also present in CD34^+ ^progenitor cells and normal peripheral blood lymphocytes [50].

In conclusion, the CD44^+ ^lymphoma cell lines expressed mainly *CD44s*, which as major splice variant of normal lymphoid tissue is believed to exert tumor suppressor functions.

### *CD44 *hypermethylated lymphoma are resistant towards anti-CD44 induced apoptosis

**Figure 6:**
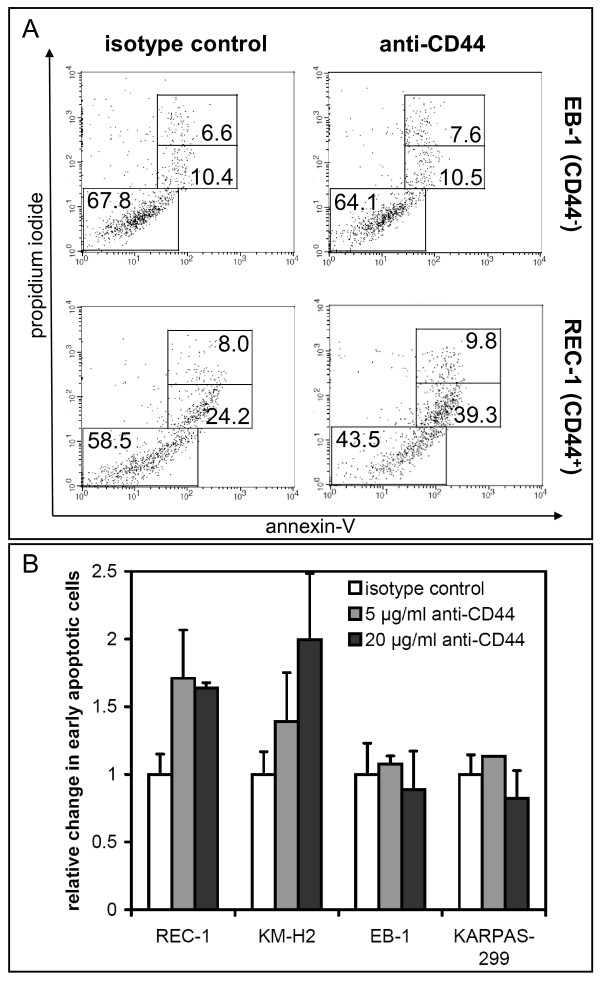
**Analysis of apoptosis after anti-CD44 ligation**. **(A) **Apoptosis was analyzed after incubation of cells for 24 h with anti-CD44 mAb (20 μg/ml) or isotope control antibody (20 μg/ml) by annexin-V/PI staining and measured by flow cytometry as shown for cell lines EB-1 (CD44^-^) and REC-1 (CD44^+^). Data are representative of three independent experiments with similar results. **(B) **Mean of early apoptotic cells (annexin-V^+^, PI^-^) in two CD44^+ ^(REC-1 and KM-H2) and two CD44^- ^(EB-1 and KARPAS-299) lymphoma cell lines after treatment with anti-CD44 in different concentrations for 24 h (n = 3). The number of early apoptotic cells in the corresponding isotope controls were set to 1. Note that anti-CD44 induced apoptosis occurred only in the CD44^+ ^cell lines.

Uncontrolled activation of oncogenes and the loss of TSG can promote sustained cell proliferation and tumorigenesis. Therefore, we decided to investigate whether the loss of *CD44 *by DNA hypermethylation in certain NHL subtypes might have functional consequences. CD44 activation by its natural ligand hyaluronic acid (HA) as well as by appropriate anti-CD44 mAbs stimulates multiple cell type-dependent CD44-associated functions, including proliferation, differentiation, migration, contact inhibition, and apoptosis [51]. In acute myeloid leukemia cell lines, ligation of cell surface CD44 with an anti-CD44 mAb can inhibit proliferation and induce apoptosis [52,53]. Therefore, CD44^+ ^and CD44^- ^lymphoma cell lines were cultivated for 24 h in the presence of an anti-CD44 mAb (G44-26) or of a matched isotope control antibody and were subsequently analyzed for induction of apoptosis via annexin-V/PI staining (Figure 6). CD44 ligation led to a considerable increase of early (annexin-V^+^/PI^-^) apoptotic cells in the CD44^+ ^cell line REC-1 (MCL), whereas the CD44^- ^cell line EB-1 (BL) was unaffected (Figure 6A). Thus, the anti-CD44 mAb triggered apoptosis in CD44^+ ^and *CD44 *unmethylated cell lines (REC-1 and KM-H2), whereas CD44^- ^lymphoma cell lines, which are *CD44 *hypermethylated, showed no response (Figure 6B). This supports a tumor suppressor function for CD44 in lymphoma. Since resistance towards apoptosis is a hallmark of most types of cancer, loss of *CD44 *due to *de novo *CpG island hypermethylation in certain lymphoma subtypes, especially observed in BL cell lines and patients may prove advantageous for the tumor cell and promote lymphomagenesis. Accordingly, it has been reported for prostate cancer that hypermethylation of the *CD44 *promoter is associated with the downregulation of *CD44 *expression during metastic progression [21].

The fact that CD44^+ ^HL and MCL cell lines were sensitive to anti-CD44 induced apoptosis may allow for new therapeutic strategies by targeting cell surface CD44. Promising clinical trials of anti-CD44 antibody drug conjugates in breast cancer and head and neck squamous cell carcinoma have already been conducted [54]. In addition, *CD44 *could become a valuable new epigenetic target in *CD44 *hypermethylated NHL for treatment with DNA methyltransferase inhibitors. Whether a combination of DNA demethylating agents and anti-CD44 mAbs might be applicable for CD44^- ^lymphoma should be an object of future studies. In CD44^- ^lymphoma cell lines Aza treatment alone induced apoptosis and a simultaneous incubation with anti-CD44 mAb showed no additive effects (data not shown).

## Conclusions

Our studies on TSG methylation patterns in lymphoma cell lines and patient samples led to the identification of novel epigenetically silenced TSG, with *CD44 *being the most interesting one. Hypermethylation of *CD44 *occurred preferentially in ALCL, BL, DLBCL and FL cell lines, whereas HL and MCL cell lines were mainly unmethylated and tested positive for CD44 mRNA and protein. Concordant with the methylation pattern observed in cell lines, *CD44 *hypermethylation was identified frequently in BL but not in MCL patients. The *CD44 *expression profiles obtained from lymphoma cell lines resemble previous reports on differential CD44 expression in diverse primary lymphoma entities [45,55]. No regulatory mechanisms for *CD44 *expression in lymphoma have been described so far. We show here, that *CD44 *expression is epigenetically regulated in lymphoma cell lines and presumably also in primary lymphoma. *CD44 *hypermethylation resulted in transcriptional silencing of the gene, which could be reactivated by treatment with the DNA demethylating agent Aza. Furthermore, ligation of cell surface CD44 with an anti-CD44 antibody triggered apoptosis in CD44^+ ^lymphoma cell lines, whereas CD44^- ^cell lines with *CD44 *hypermethylation were resistant towards anti-CD44 induced apoptosis. These experiments support the tumor suppressor function of CD44 in lymphoma. Therefore, epigenetic silencing of *CD44 *might be advantageous during lymphomagenesis and should be considered in further investigations. In the future, CD44 should be evaluated as a potential target for epigenetic therapy and as a target for antibody-based therapies in CD44^+ ^lymphoma subtypes.

## Competing interests

The authors declare that they have no competing interests.

## Authors' contributions

SE designed the study, performed experiments and data analyses and wrote the manuscript. BS carried out data analyses. AR, EMH and RS provided patient samples and performed DNA extractions thereof. JR made MS-MLPA analyses. MZ performed flow cytometry. HGD provided cell lines and good advice. HQ supervised the project. All authors read and approved the final manuscript.

## Pre-publication history

The pre-publication history for this paper can be accessed here:

http://www.biomedcentral.com/1471-2407/10/517/prepub

## Supplementary Material

Additional file 1 **Methylation status and deletions of TSG in tumor samples of 29 BL, 10 DLBCL and 11 MCL patients**. MS-MLPA results of primary lymphoma samples show the methylation status and copy number of 24 TSG. TSG are sorted from left to right according to the order of Figure 1. Yellow = TSG unmethylated and not deleted; green = TSG methylated and/or deleted; del = deleted; o. del = only deleted, not methylated; nd = not determined. Click here for file

Additional file 2 ***CD44 *variant analysis in CD44^+ ^lymphoma cell lines**. **(A) **Reverse transcriptase PCR was performed with primers (blue arrows) flanking all variant exons (colored exons in *CD44 *exon structure) of *CD44 *to examine expression of *CD44 *splice variants. **(B) **The agarose gel shows that the *CD44s *PCR product (142 bp) was the main variant present in the CD44^+ ^lymphoma cell lines and PBMC (peripheral blood mononuclear cells). A second noticeable PCR product turned out to be the splice variant *CD44v10 *after sequencing analysis. As expected, CD44^- ^cell lines (NAMALWA, HT) tested negative. Click here for file
