# Supplementary material for: Epigenetic regulation of CD44 in Hodgkin and non-Hodgkin lymphoma
Source: BMC Cancer. 2010 Sep 29;10:517. doi: 10.1186/1471-2407-10-517 (PMC2955612; doi:10.1186/1471-2407-10-517)
Supplement: Additional file 1 — Methylation status and deletions of TSG in tumor samples of 29 BL, 10 DLBCL and 11 MCL patients. MS-MLPA results of primary lymphoma samples show the methylation status and copy number of 24 TSG. TSG are sorted from left to right according to the order of Figure 1. Yellow = TSG unmethylated and not deleted; green = TSG methylated and/or deleted; del = deleted; o. del = only deleted, not methylated; nd = not determined. [file 1471-2407-10-517-S1.PDF]

Additional File 1

		DAPK1	RARB	CDH13	IGSF4	TIMP3	ESR1	CD44	TP73	FHIT	CHFR	CDKN2B	CDKN2A	RASSF1	APC	HIC1	CASP8	PTEN	GSTP1	ATM	BRCA2	MLH1	CDKN1B	BRCA1	VHL
BL	BL1														nd										
	BL2														nd										
	BL3														nd	o. del (1n)									
	BL4														nd										
	BL5														nd										
	BL6														nd										
	BL7														nd										
	BL8														nd										
	BL9	o. del (1n)													nd				o. del (1n)						
	BL10														nd										
	BL11	del (1n)		del (1n)											nd	o. del (1n)									
	BL12														nd										
	BL13														nd										
	BL14														nd										
	BL15	del (1n)													nd										
	BL16														nd										
	BL17		o. del (1n)												nd										
	BL18	del (1n)													nd										
	BL19	del (1n)													nd										
	BL20	o. del (1n)													nd										
	BL21														nd										
	BL22	o. del (1n)													nd										
	BL23		o. del (1n)												nd										
	BL24		o. del (1n)												nd										
	BL25														nd										
	BL26														nd										
	BL27	o. del (1n)													nd										
	BL28														nd										
	BL29	o. del (1n)													nd	o. del (1n)									
DLBCL	DLBCL1																				o. del (1n)				
	DLBCL2																								
	DLBCL3																								
	DLBCL4																								
	DLBCL5		del (1n)			del (1n)			o. del (1n)																
	DLBCL6																								
	DLBCL7						o. del (1n)					o. del (1n)	o. del (1n)									o. del (1n)			
	DLBCL8	del (1n)										o. del (1n)													
	DLBCL10																								
	DLBCL11																								
MCL	MCL1	o. del (1n)																			o. del (1n)				
	MCL2																	o. del (1n)			o. del (1n)				
	MCL3											o. del (1n)													
	MCL4				o. del (1n)																				
	MCL5				o. del (1n)		o. del (1n)																		
	MCL6																								
	MCL7						o. del (1n)																		
	MCL8	o. del (1n)			o. del (1n)															o. del (1n)					
	MCL9				o. del (1n)															o. del (1n)					
	MCL10																								
	MCL11																								
