# Supplementary figures and images for: Epigenetic regulation of CD44 in Hodgkin and non-Hodgkin lymphoma
Source: BMC Cancer. 2010 Sep 29;10:517. doi: 10.1186/1471-2407-10-517 (PMC2955612; doi:10.1186/1471-2407-10-517)

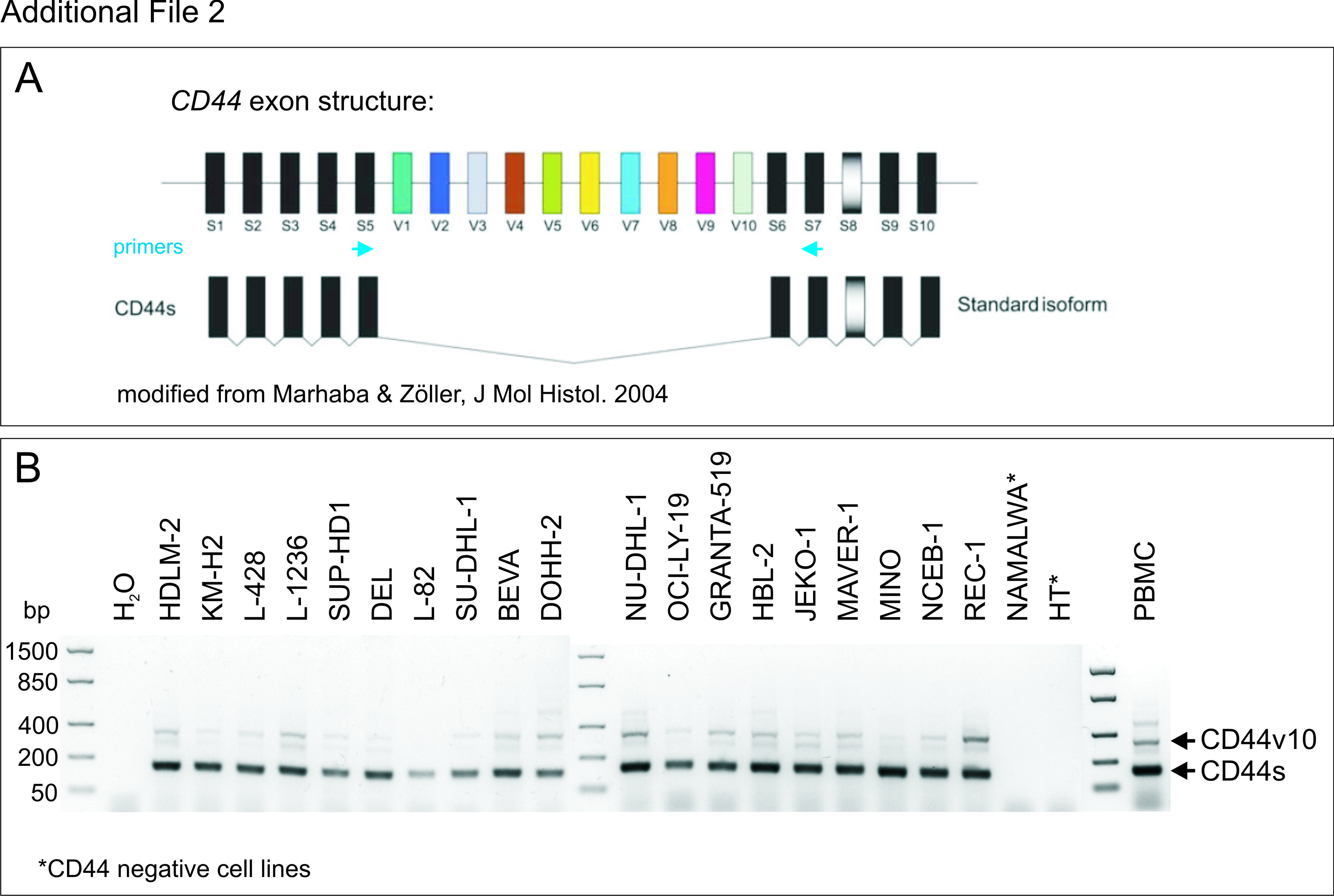

Supplement: Additional file 2 — CD44 variant analysis in CD44+ lymphoma cell lines. (A) Reverse transcriptase PCR was performed with primers (blue arrows) flanking all variant exons (colored exons in CD44 exon structure) of CD44 to examine expression of CD44 splice variants. (B) The agarose gel shows that the CD44s PCR product (142 bp) was the main variant present in the CD44+ lymphoma cell lines and PBMC (peripheral blood mononuclear cells). A second noticeable PCR product turned out to be the splice variant CD44v10 after sequencing analysis. As expected, CD44- cell lines (NAMALWA, HT) tested negative. [file 1471-2407-10-517-S2.JPEG]
